# Management of Endothelial Dysfunction in Systemic Sclerosis: Current and Developing Strategies

**DOI:** 10.3389/fmed.2021.788250

**Published:** 2021-12-22

**Authors:** Djúlio César Zanin-Silva, Maynara Santana-Gonçalves, Marianna Yumi Kawashima-Vasconcelos, Maria Carolina Oliveira

**Affiliations:** ^1^Center for Cell-Based Therapy, Regional Hemotherapy Center of the Ribeirão Preto Medical School, University of São Paulo, Ribeirão Preto, Brazil; ^2^Basic and Applied Immunology Graduate Program, Ribeirão Preto Medical School, University of São Paulo, Ribeirão Preto, Brazil; ^3^Oncology, Stem Cell and Cell-Therapy Graduate Program, Ribeirão Preto Medical School, University of São Paulo, Ribeirão Preto, Brazil; ^4^Internal Medicine Graduate Program, Ribeirão Preto Medical School, University of São Paulo, Ribeirão Preto, Brazil; ^5^Department of Internal Medicine, Ribeirão Preto Medical School, University of São Paulo, Ribeirão Preto, Brazil

**Keywords:** systemic sclerosis, vasculopathy, cellular therapy, endothelial cells, vasodilator agent

## Abstract

Systemic Sclerosis (SSc) is an autoimmune disease marked by dysregulation of the immune system, tissue fibrosis and dysfunction of the vasculature. Vascular damage, remodeling and inadequate endothelial repair are hallmarks of the disease. Since early stages of SSc, damage and apoptosis of endothelial cells (ECs) can lead to perivascular inflammation, oxidative stress and tissue hypoxia, resulting in multiple clinical manifestations. Raynaud's phenomenon, edematous puffy hands, digital ulcers, pulmonary artery hypertension, erectile dysfunction, scleroderma renal crisis and heart involvement severely affect quality of life and survival. Understanding pathogenic aspects and biomarkers that reflect endothelial damage in SSc is essential to guide therapeutic interventions. Treatment approaches described for SSc-associated vasculopathy include pharmacological options to improve blood flow and tissue perfusion and, more recently, cellular therapy to enhance endothelial repair, promote angiogenesis and heal injuries. This mini-review examines the current knowledge on cellular and molecular aspects of SSc vasculopathy, as well as established and developing therapeutic approaches for improving the vascular compartment.

## Introduction

Systemic sclerosis (SSc) is an autoimmune disease marked by diffuse vasculopathy, immunological dysregulation and fibrosis of the skin and internal organs. Vascular manifestations derive mostly from impaired blood flow and tissue ischemia, and are a challenge for the management of SSc patients (1–3). In this mini-review, we examine the current and developing therapeutic interventions with pharmacological agents and cellular therapy for SSc-associated vasculopathy.

## Pathophysiology of the Vascular Endothelium in Systemic Sclerosis

**Figure 1:**
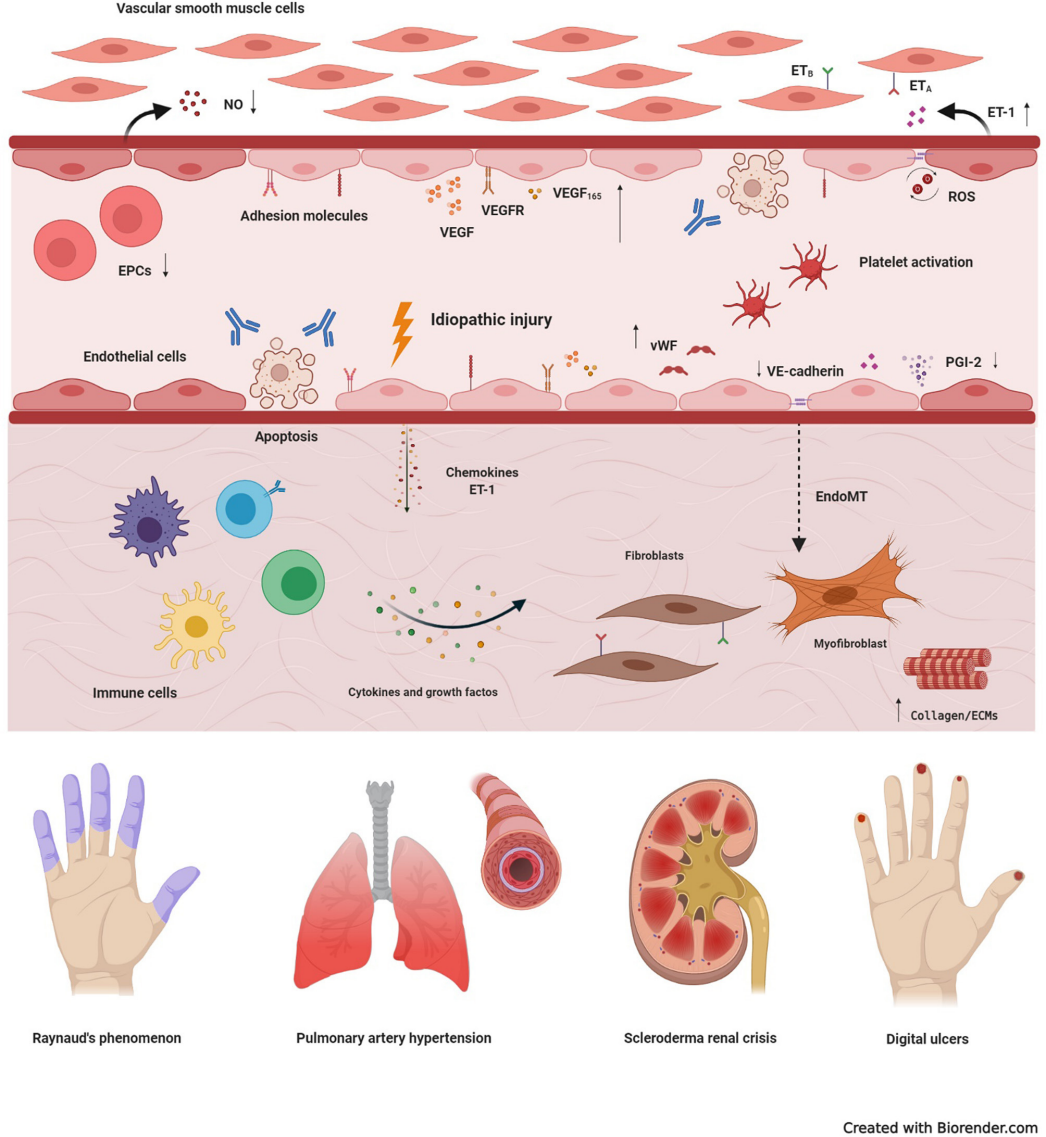
Basic mechanisms of systemic sclerosis-related vasculopathy. Vascular injury is considered an initial event in the development of systemic sclerosis (SSc), and may be triggered by multiple factors, including autoantibodies, infectious agents, reactive oxygen species (ROS), or idiopathic stimuli. In the early stages of disease, vascular damage leads to activation of endothelial cells (ECs), with expression of adhesion molecules, production of chemokines, von Willebrand factor (vWF) and vasoconstrictor agents, such as endothelin-1 (ET-1). Molecules produced by the injured endothelium recruit immune cells, that generate a perivascular infiltrate. Prolonged inflammation leads to tissue fibrosis, with excessive activation of resident fibroblasts that transdifferentiate into myofibroblasts, the main cell type involved in excessive collagen production and other extracellular matrix components (ECMs). Myofibroblasts are also originated through the endothelial-to-mesenchymal transition (EndoMT). Dysfunction of endothelial progenitor cells (EPCs), antibody-induced ECs apoptosis, persistent platelet activation, decreased production of vasodilatory nitric oxide (NO) and prostaglandin I-2 (PGI-2), synthetized by ECs, also participate in the pathogenesis of SSc-vasculopathy. In addition, compensatory mechanisms of vasculogenesis and angiogenesis, including vascular endothelial growth factor (VEGF) and its receptor (VEGFR), are dysregulated and ineffective. High expression of VEGF165, an anti-angiogenic isoform, contributes to this scenario. Reactive oxygen species, further contribute to intensify damage and activation of the endothelium and, thus, increase tissue injury. Clinical manifestations of SSc-related vasculopathy include Raynaud's phenomenon, pulmonary arterial hypertension, scleroderma renal crisis, telangiectasias, digital ulcers and digital pitting scars, which severely affect quality of life and may compromise survival. ET_A_, type A endothelin receptor; ET_B_, type B endothelin receptor.

The endothelium is a metabolically active tissue that ensures regulation of vascular tone, coagulation and fibrinolysis, smooth muscle proliferation, cell adhesion and inflammation (4). Vascular injury is an early event in SSc, with damage and activation of endothelial cells (ECs) (5, 6) (Figure 1). Injured ECs in SSc produce increased levels of endothelin-1 (ET-1) and von Willebrand factor (vWF), and low levels of nitric oxide (NO) and endothelial nitric oxide synthase (5). The resulting imbalance between vasodilation and vasoconstriction modifies the vascular tone, contributing to tissue hypoxia. ET-1 also induces differentiation of fibroblasts into a myofibroblastic phenotype, promoting intimal hyperplasia, luminal narrowing, and vessel obliteration (7, 8). Myofibroblasts may also be originated through the endothelial-to-mesenchymal transition (EndoMT) (9), when ECs downregulate expression of markers such as CD31 and VE-cadherin, and assume a myofibroblast phenotype, characterized by fusiform morphology and expression of α-SMA (10). The abnormal vascular tonus and the increased expression of vWF stimulate platelet aggregation and hypercoagulation, leading to further vascular damage (11, 12). Reactive oxygen species contribute to further enhance the damage, participating in the initiation and progression of SSc (2, 5).

Cell adhesion molecules play an important role in promoting endothelial integrity, besides regulating leukocyte migration, vascular permeability and angiogenesis (13). Increased expression of adhesion molecules and their soluble levels, detected in early stages of SSc, correlate with disease severity and visceral involvement (14–18). Indeed, increased levels of E-selectin, vascular cell adhesion molecule 1 (VCAM-1) and intercellular adhesion molecule 1 (ICAM-1) lead to activation of ECs, dysregulation of angiogenesis and, as consequence, chronic and progressive vascular damage (19).

## Impaired Compensatory Angiogenesis and Vasculogenesis

In SSc, damage and apoptosis of ECs result in loss of capillaries that are not repaired by compensatory mechanisms of vasculogenesis and angiogenesis (20, 21). Vascular endothelial growth factor (VEGF) regulates blood vessel growth, with key role in the process of angiogenesis (22). Serum levels of VEGF and its receptor (VEGFR) are increased in SSc (16, 23–26). Exposure to high levels of VEGF causes an exaggerated angiogenic stimulus, with proliferation of ECs, resulting in chaotic architecture of vessels, as observed by capillaroscopy (19). An anti-angiogenic isoform, VEGF165, has been described in SSc patients (27), and platelet releases containing VEGF165 impair angiogenesis *in vitro* (28). In addition, function and frequencies of endothelial progenitor cells (EPCs) are compromised in SSc, playing a defective role in vasculogenesis (29). Table 1 describes additional biomarkers associated with vascular damage in SSc.

**Table 1:** Biomarkers associated with endothelial activation or vascular damage in SSc and clinical correlates.

**Biomarkers**	**Class/function**	**Clinical associations**	**References**
Adhesion molecules (ICAM-1, VCAM-1, selectins)	Cell–cell interactions	Capillaroscopic abnormalities Disease severity Pulmonary fibrosis	(14, 15, 18, 30–35)
Angiopoietin system (ANG-Tie)	Angiogenesis	Disease activity Digital ulcers Esophageal dysmotility Microangiopathy Proliferative vasculopathy	(36–41)
Anti-centromere (ACA)	Autoantibodies	Microangiopathy Pulmonary arterial hypertension	(41–43)
Anti-AT1R and -ETAR	Autoantibodies	Digital ischemic Pulmonary arterial hypertension (PAH)	(44, 45)
Anti-endothelial cell (AECA)	Autoantibodies	Pulmonary fibrosis	(46)
Anti-RNA polymerase III	Autoantibodies	Gastric Antral Vascular Ectasia (GAVE) Scleroderma renal crisis Diffuse skin thickening Cardiopulmonary involvement Rapid disease progression	(34, 47–55)
Anti - topoisomerase I (anti-SCl70)	Autoantibodies	Digital ulcers Heart involvement Interstitial lung disease	(56)
Endoglin (CD105)	Type I membrane glycoprotein.	Digital ulcers	(57)
Endothelin-1	Vasoconstrictor molecule	Interstitial lung disease Right ventricle dysfunction	(58–62)
Endostatin	Angiogenesis	Digital vascular damage Skin and pulmonary fibrosis	(63, 64)
Thrombomodulin	Coagulation	Pulmonary hypertension	(65)
Thrombospondin-1 (TSP-1)	Antiangiogenic glycoprotein	Brachio-cervical inflammatory myopathy	(66)
Vascular endothelial cell growth (VEGF)	Angiogenesis	Diffuse skin subset Interstitial lung involvement Nailfold capillary loss Pulmonary Artery Hypertension (PAH)	(25, 67–72)

*ETAR, endothelin receptor type A; AT1R, Ang receptor type-1*.

## Clinical Manifestations of SSc-Associated Vasculopathy

Raynaud's phenomenon is one of the first manifestations of the disease (8, 73). Progressive structural damage of the vessels, followed by proliferative endarteritis and consequent tissue ischemia, leads to systemic involvement, characterizing SSc as a microvascular disease. Telangiectasias and digital ulcers are frequent vascular manifestations of SSc, and associate with poor prognosis (74, 75). Scleroderma renal crisis, a severe clinical condition characterized by poor renal cortical perfusion and rapidly progressive renal failure, was a leading cause of death until the 1970s, when use of angiotensin-converting enzyme inhibitors significantly improved patient management and outcomes (76–80). Primary and secondary cardiac involvements are described as frequent and probably underestimated in SSc (81–83), and from 5 to 15% of SSc patients develop pulmonary hypertension (79, 81). Less explored, but still frequent vascular manifestations of SSc are erectile dysfunction, vascular malformations of the gastro-intestinal mucosa (gastric antral vascular ectasia - GAVE) and, to some extent, myopathy (66, 84–86). Routine assessments for vascular involvement include clinical inspections, evaluation of organ function and, when required, right-heart catheterism. Such manifestations should be actively investigated and treated early, before advanced organ damage.

## Pharmacological Approaches

**Figure 2:**
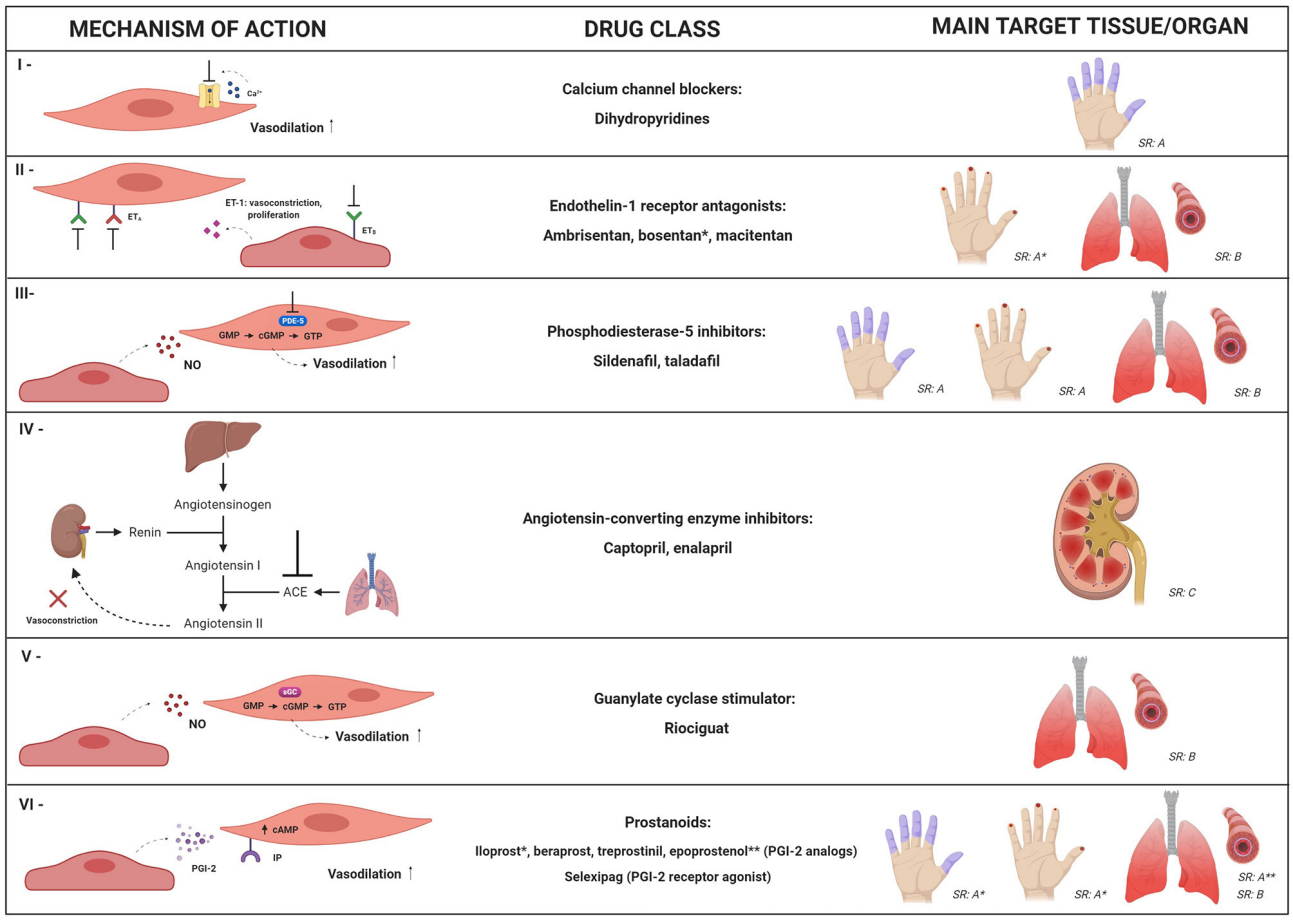
Mechanistic targets of the main pharmacological approaches for systemic sclerosis-related vasculopathy. Multiple drug options, with different mechanistic approaches, can be used for management of vascular manifestations in SSc patients. This figure summarizes mechanisms and clinical indications for: calcium channel blockers (I); endothelin-1 receptor antagonists (II); phosphodiesterase-5 (PDE-5) inhibitors (III); angiotensin-converting enzyme (ACE) inhibitors (IV); guanylate cyclase stimulator – riociguat (V) and prostanoids (VI). SR: strength of recommendation; SR: *A*, SR: *B* or SR: *C* according to the European League against Rheumatism, in 2017 (87). Dihydropyridines, PDE-5 inhibitors and iloprost are recommended for SSc-associated Raynaud's phenomenon (SR: *A*); bosentan is recommended to reduce the number of new digital ulcers (SR: *A*); PDE-5 inhibitors and iloprost are recommended for active digital ulcers (SR: *A*); PDE-5 inhibitors, endothelin-1 receptor antagonists (bosentan, ambrisentan, and macitentan) and riociguat are recommended for treatment of SSc-related pulmonary artery hypertension (SR: *B*); epoprostenol (SR: *A*) and other prostacyclin analogs (SR: *B*) are recommended for severe SSc-related pulmonary artery hypertension; ACE inhibitors are recommended as treatment for scleroderma renal crisis (SR: *C*). ^*^ and ^**^ indicate clinical conditions in which specific drugs from the class are recommended. NO, nitric oxide; ET-1, endothelin-1; ET_A_, type A endothelin receptor; ET_B_, type B endothelin receptor; cGMP, cyclic guanosine-5-monophosphate; sGC, soluble guanylate cyclase; cAMP, cyclic adenosine monophosphate; GTP, guanosine-5′-triphosphate; IP, prostacyclin receptor; PGI-2, prostaglandin I-2.

Therapeutic strategies for vasculopathy in SSc aim to improve symptoms of Raynaud's phenomenon (RP), heal and prevent development of digital ulcers (DU), and decrease the ischemic damage to internal organs. Multiple pharmacological options, with different mechanistic approaches, are available and recommended in the management of SSc patients (Figure 2) (87). New strategies, including cell therapy, have been developed to further improve this aspect of the disease.

## Calcium Channel Blockers

Calcium channel blockers reduce intracellular calcium concentrations, inducing relaxation of smooth muscle and vasodilation (88). Dihydropyridines are broadly recommended to attenuate severity and frequency of uncomplicated RP in SSc (87, 89). Short and long-term use of calcium channel blockers decreased plasma markers of oxidative stress (90), and *in vitro*, nicardipine protected ECs against oxidative injury (91). Calcium channel blockers also decreased serum concentrations of N-terminal pro-brain natriuretic peptide (NT-proBNP) in patients with SSc-associated PAH, indicating a possible antispastic and vasodilatory effect on the pulmonary circulation, not corroborated, however, by hemodynamic changes (92). In patients with <5 years of SSc, nifedipine and nicardipine improved myocardial perfusion and left ventricle function, respectively, supporting the hypothesis of myocardial Raynaud's phenomenon in SSc (93).

## Endothelin-1 Receptors Antagonists

Endothelin-1 receptor antagonists target ET-1, a crucial mediator in SSc vasculopathy. Ambrisentan is a selective type A endothelin receptor antagonist, while bosentan and macitentan are dual antagonists, targeting both type A and B receptors (88). In two randomized clinical trials, bosentan prevented the development of new DU, but did not heal active DU (94, 95). Ambrisentan reduced the number of active and new DU in SSc patients, also decreasing pain and disability (96, 97).

Bosentan and ambrisentan improved hemodynamic parameters in patients with SSc-PAH (98, 99). Bosentan also decreased serum concentrations of endothelial activation markers ICAM-1, VCAM-1, P-selectin and PECAM-1 (100). *In vitro* experiments with preincubation of microvascular endothelial cells (MVECs) from SSc patients with bosentan or macitentan decreased the expression of mesenchymal markers, identifying a possible pharmacological interference pathway to prevent EndoMT (101).

## Phosphodiesterase-5A Inhibitors

Phosphodiesterase-5A (PDE-5A) hydrolyzes the cyclic guanosine-5-monophosphate (cGMP), associated to the nitric oxide (NO) vasodilator pathway. PDE-5A inhibitors reduce the metabolism of cGMP, intensifying the vasodilatory effects of NO (102). In SSc patients, PDE-5A inhibitors decreased frequency and duration of RP attacks, improved DU healing (103) and reduced disability and discomfort associated with RP (104). For SSc-PAH, sildenafil reduced pulmonary artery pressure, with beneficial effects on cardiopulmonary status (105). Combined therapy of tadalafil plus ambrisentan resulted in better responses for SSc-PAH than monotherapy with either agent (106). However, sildenafil did not affect the number of circulating EPCs or VEGF serum levels in SSc patients with vasculopathy (107–109). PDE-5 inhibitors have been also investigated as treatment for erectile dysfunction and, although SSc patients have poor response to on-demand administration, daily fixed doses may be effective (110).

## Prostanoids

Prostacyclin, also known as prostaglandin I-2 (PGI-2), is synthetized by vascular ECs, promoting vasodilation and decreasing platelet aggregation, inflammation and vascular smooth muscle proliferation (111). Prostacyclin analogs (iloprost, beraprost, treprostinil, and epoprostenol) and the prostacyclin receptor agonist (selexipag) are available pharmaceutical agents that enhance the prostacyclin pathway and thus promote vasodilation (88).

Iloprost was effective for treatment of RP, DU and PAH in SSc patients (112–117), also decreasing serum levels of ICAM-1, VCAM-1 and E-selectin, reflecting reduced activation of ECs (115). Iloprost and bosentan combinatory therapy increased the number of nailfold capillaries (118). Beraprost did not prevent development of DU (119) and had little effect on hemodynamic parameters in SSc-PAH (120). Conversely, epoprostenol improved clinical status and hemodynamic parameters (121, 122), and increased serum levels of adiponectin (123), suggesting effects on vascular function (124) and on adipose tissue metabolic pathways (123). Treprostinil improved cutaneous blood flow (125, 126) and healing of DU (127), but recent studies failed to show changes in vascular, angiogenic and inflammatory biomarkers (128).

Prostacyclin agonists have short half-life, high frequency of administration and multiple side effects, and products with more convenient posology have been investigated. Selexipag is an oral selective prostacyclin receptor agonist that promotes vasodilation by increasing cyclic adenosine monophosphate concentrations (129) and has been effective for PAH (130). For the peripheral circulation, however, efficacy of this drug is still debated. While in a randomized, placebo-controlled study, selexipag failed to reduce the frequency of RP attacks (131), an open observational study showed considerable improvement of RP, also suggesting that selexipag may be effective for DU healing and resolution of DU related-pain (132).

## Angiotensin-Converting Enzyme Inhibitors

Angiotensin-converting enzyme (ACE) inhibitors block the conversion of angiotensin I into the vasoconstrictor agent angiotensin II, promoting rapid control of the blood pressure (133, 134). Over the past decades, ACE inhibitors had a significant impact on outcomes of SSc patients with scleroderma renal crises (SRC), decreasing the need for dialysis and increasing survival (78, 135). Prophylactic use, however, did not reduce the incidence and was associated with poor prognosis and risk of death after onset of SRC (136, 137).

## Riociguat

Riociguat is a soluble guanylate cyclase (sGC) stimulator that leads to strong vasodilator effects on the pulmonary arteries (138–144). Clinical trials in PAH patients, including SSc, showed improvements in pulmonary vascular resistance (145). An initial study failed to demonstrate significant reduction of active or painful DU, or changes in plasma levels of VEGF, E-selectin, VCAM-1 and ICAM-1, but long-term observations detected complete healing of the DU (146), and improvement of discomfort and disability associated with RP (147). Larger studies should determine the impact of riociguat on the peripheral vasculature (148, 149).

## Cyclophosphamide

Cyclophosphamide (CYC), an immunosuppressive drug mostly used for SSc-related interstitial lung disease (150), also affects the vascular compartment, both in experimental and clinical scenarios (5). Cyclophosphamide improved nailfold capillaroscopic patterns (151), increased the number of circulating EPCs and reduced serum levels of VEGF, E-selectin and thrombomodulin, markers of endothelial injury and activation (152, 153), indicating that CYC may affect pathogenic processes associated with lung damage and fibrosis, such as re-endothelialization and re-epithelialization of the alveolar-capillary barrier (154).

Dermal MVECs exposed to the serum of CYC-treated SSc patients had better proliferation and less apoptosis than those exposed to serum of untreated SSc patients. Additionally, serum levels of antiangiogenic factors pentraxin 3 (PTX3), matrix metalloproteinase (MMP)-12, endostatin and angiostatin were significantly reduced after CYC treatment in SSc patients, suggesting a therapeutic effect on peripheral microvasculopathy (155).

## Fluoxetine

Fluoxetine is a selective serotonin reuptake inhibitor that has been recommended as treatment for SSc RP attacks (87). Serotonin participates in Raynaud's phenomenon pathogenesis as a stimulator (156–158), but fluoxetine has paradoxical vasodilation effects, mediated by 5HT7 and 5HT2B receptors (159), that affect the NO and calcium pathways (160–162). Fluoxetine reduced the severity of RP attacks in SSc patients, with no impact on soluble P-selectin or wWF levels, however (163).

## Less Traditional Therapeutic Interventions

Statins have been studied in immune-mediated diseases, including SSc, due to their immunomodulatory effects (164–166). Rosuvastatin improved endothelial function in SSc patients, assessed by skin microcirculation and brachial artery flow (167). Atorvastatin improved the visual analog scale for RP and DU, and was associated with reduced plasma levels of endothelial activation markers ICAM-1, E-selectin and ET-1, oxidative stress and vWF activity (159, 168). Atorvastatin led to transient increase in numbers of circulating EPCs (159), but failed to induce maturation of EPCs into ECs *in vitro*, indicating a limited therapeutic potential on vascular repair (169). Topical nitrate application is also effective in the treatment of RP in SSc patients. Nitrates are degraded into NO, increase cGMP concentration in the vascular smooth muscle and lead to vasodilation (170). Nitroglycerine tapes improved the peripheral circulation in SSc patients (171). Likely, MQX-503, a novel compound of nitroglicerine, was well-tolerated, improving the cutaneous blood flow in SSc patients (172). Topical application of glyceryl trinitrate increased DU perfusion, indicating supplementation of the NO pathway by nitrates as a promising strategy (173).

More recently, pirfenidone, an antifibrotic drug considered for treatment of interstitial lung disease (174), has shown vasodilatory effects. In animal models, pirfenidone induced pulmonary artery relaxation, restored renal blood flow and stimulated the NO pathway involving voltage-gated KV7 channels (175, 176). Clinical studies should further evaluate potential effects of the drug on the vascular compartment.

Local therapies are also described for SSc-associated vasculopathy. Botulinum toxin (Btx) inhibits acetylcholine release from presynaptic nerve terminals, reducing vascular smooth muscle contraction, and improving local circulation (177). A randomized controlled trial was inconclusive, since administration of Btx unexpectedly worsened blood flow in hands of SSc patients with RP, but patient perceptions of hand function and discomfort improved (178). Series of cases and one systematic review show healing of DU and reduction of pain in most patients after digital Btx applications (179, 180). Laser and intense pulsed light therapies have been investigated for digital ulcers and telagiectasies, with reports of safety and improvements of patient perception and blood flow (181, 182). In SSc patients with severe ischemic complications, especially vascular obstruction of the hands, peripheral or digital sympathectomy, microsurgical revascularization and digital artery reconstruction may be indicated. Besides limitations, these approaches are able to increase blood perfusion, decrease or eliminate pain, and may be recommended for selected cases (183).

## Cellular Therapies for SSc-Associated Vasculopathy

In the last two decades, different cellular therapy approaches have been investigated for SSc patients (184). Local applications of fat graft/adipose-derived stem cells (ADSCs) or bone marrow hematopoietic stem cells show the strongest potential for regeneration of damaged tissue and vascular remodeling.

## Fat Grafting and Stromal Vascular Fraction/Adipose-Derived Stem Cells-Based Therapy

Adipose-derived stem cells can be isolated from the stromal vascular fraction (SVF), located in the white adipose tissue (184), and show robust angiogenic activity (185–195). Patients with SSc treated with local administration of autologous fat grafts showed improvement of RP symptoms (188, 195), and complete healing of DU (189, 193). Treatment also led to significant increase of capillary density in fingers affected by DU (193) and enabled better pain control (189). Furthermore, autologous fat grafts increased mouth opening and vascularization in perioral areas of SSc patients (191).

Local injections of autologous SVF also improved RP, vascular flow, hand pain and finger edema in SSc patients (190, 192). Combination of autologous SVF and platelet-rich plasma, which is reported to enhance ADSC proliferation (194), also increased capillary density and decreased vascular ectasia in SSc patients, suggesting induction of neoangiogenesis (196). When locally implanted, ADSCs secrete VEGF and fibroblast growth factor, which may support local angiogenesis (197). These cells promote proliferation and inhibit apoptosis of ECs (198). Nevertheless, ADSCs isolated from SSc patients exhibit abnormal proliferation, metabolism, differentiation potential, and have a pro-fibrotic phenotype (194, 199–201), suggesting that despite beneficial effects, autologous ADSCs may not achieve full potential in tissue repair (185). More efforts are needed to investigate how they interfere with disease pathogenesis, and if there is potential for systemic therapy (185).

## Hematopoietic Stem Cell Transplantation

Over the past 25 years, hundreds of patients with severe and progressive SSc have undergone autologous stem cell transplantation (AHSCT) (202), with better outcomes regarding survival, disease control and quality of life, when compared to conventional treatment (203–206). Indications for AHSCT include mainly fibrosis-related manifestations of SSc, such as skin thickening and interstitial lung disease (202–206). Patients with severe vascular manifestations, especially those with pulmonary hypertension or scleroderma renal crisis are usually excluded (143–147) and extensive cardiac assessment is recommended to avoid inclusion of patients with asymptomatic cardiac involvement (207). The procedure resets the immune system and promotes better control of autoreactivity, inflammation and fibrosis processes (208, 209).

To date, little is known about the impact of AHSCT on SSc-associated vasculopathy. Stem cell transplantation did not change dermal vessel density evaluated by immunostaining for endothelial markers CD31, VE-cadherin and vWF (210). On the other hand, AHSCT partially restored the microvascular structure assessed by nailfold video capillaroscopy (211), increased capillary counts, normalized cutaneous expression of VE-cadherin and decreased the expression of Interferon α mRNA in the skin, which is known as a potent inhibitor of angiogenesis (212, 213). Serum levels of VEGF decreased after AHSCT (214), which can be interpreted as a good result, since disrupted VEGF upregulation is associated with abnormal vessel morphology in SSc (24). Mechanisms to possibly explain the positive influence of AHSCT on the vascular compartment of SSc patients include removal of cells associated with inhibitory effects on endothelial repair, mobilization of endothelial progenitor cells from the bone marrow (212), and other still unidentified mechanisms of angiogenesis (211).

## Other Cell Types Used for SSc-Vasculopathy: Bone Marrow Mesenchymal Stromal Cells

Mesenchymal stromal cells (MSC) are potential tools to treat vascular dysfunction, due to their immunosuppressive, anti-fibrotic and proangiogenic properties (215–217). Although MSCs from SSc patients display reduced capacity to differentiate into ECs *in vitro* (218), intramuscular injections of autologous MSCs reduced necrotic areas in one SSc patient with critical limb ischemia (219). After treatment, angiographies showed important revascularization, and histological analyses showed strong expression of angiogenic factors possibly effective through paracrine mechanisms. A SSc patient with multiple active skin ulcers was treated with intravenous infusion of allogeneic MSCs, with improvement of pain and blood flow in hands and fingers (220). In five SSc patients treated with intravenous allogeneic MSC infusions, there was healing of skin ulcers, and two of these patients also healed lesions of acral necrosis (221). An ongoing double-blind randomized placebo-controlled trial aims to evaluate safety and potential efficacy of intramuscular injections of allogeneic MSC as treatment for DU. In addition to clinical evaluations, such as DU healing and hand function, this study plans also analyze biomarkers in peripheral blood and skin biopsies (222).

## Conclusions and Future Directions

Treatment of SSc-related vasculopathy remains difficult, despite the multiple available therapeutic options and targeted pathways. So far, patients seem to present advanced vascular involvement since early periods of disease, with vessel disruption and ischemic lesions. The narrow therapeutic window, associated with multiple pathophysiological presentations, makes development of new strategies a challenge. There are no reliable biomarkers of vascular severity or extension, so identification of patients with disabling or life-threatening vascular involvement is often too late. Best therapeutic effects include healing of ulcers and improvement of blood flow in pulmonary, renal and peripheral vascular beds. Cell therapy has an important potential, and may be expanded and refined in the future to achieve more substantial goals. Besides subsiding inflammation, future strategies should aim to fully repair and reverse established vascular damage.

## Author Contributions

DZ-S and MS-G conceived the study. DZ-S, MS-G, and MK-V searched the literature and wrote the draft. DZ-S created the images. MO critically revised the final version of the manuscript and provided funding. All authors contributed to the article and approved the submitted version.

## Funding

This work was supported by the Coordenação de Aperfeiçoamento de Pessoal de Nível Superior (CAPES, finance code 001 and processes 88887.598001/2021-00 and 88887.597494/2021-00), by the São Paulo Research Foundation (FAPESP) (n°2013/08135-2 and 2017/09420-3), and the Fundação de Apoio ao Ensino, Pesquisa e Assistência (FAEPA).

## Conflict of Interest

The authors declare that the research was conducted in the absence of any commercial or financial relationships that could be construed as a potential conflict of interest.

## Publisher's Note

All claims expressed in this article are solely those of the authors and do not necessarily represent those of their affiliated organizations, or those of the publisher, the editors and the reviewers. Any product that may be evaluated in this article, or claim that may be made by its manufacturer, is not guaranteed or endorsed by the publisher.
